# Inoculation of *Bacillus sphaericus* UPMB-10 to Young Oil Palm and Measurement of Its Uptake of Fixed Nitrogen Using the ^15^N Isotope Dilution Technique

**DOI:** 10.1264/jsme2.ME11309

**Published:** 2012-03-23

**Authors:** Fitri Abdul Aziz Zakry, Zulkifli H. Shamsuddin, Khairuddin Abdul Rahim, Zin Zawawi Zakaria, Anuar Abdul Rahim

**Affiliations:** 1 Faculty of Agriculture, Universiti Putra Malaysia, Malaysia; 2 Faculty of Agriculture and Food Sciences, Universiti Putra Malaysia Bintulu Campus, Malaysia; 3 Agrotechnology and Biosciences Division, Malaysian Nuclear Agency, Malaysia; 4 Biology Division, Malaysian Palm Oil Board, Malaysia

**Keywords:** biological nitrogen fixation, *Elaeis guineensis* Jacq., inoculation, ^15^N isotope dilution, PGPR

## Abstract

There are increasing applications of diazotrophic rhizobacteria in the sustainable agriculture system. A field experiment on young immature oil palm was conducted to quantify the uptake of N derived from N_2_ fixation by the diazotroph *Bacillus sphaericus* strain UPMB-10, using the ^15^N isotope dilution method. Eight months after ^15^N application, young immature oil palms that received 67% of standard N fertilizer application together with *B. sphaericus* inoculation had significantly lower ^15^N enrichment than uninoculated palms that received similar N fertilizers. The dilution of labeled N served as a marker for the occurrence of biological N_2_ fixation. The proportion of N uptake that was derived from the atmosphere was estimated as 63% on the whole plant basis. The inoculation process increased the N and dry matter yields of the palm leaflets and rachis significantly. Field planting of young, immature oil palm in soil inoculated with *B. sphaericus* UPMB-10 might mitigate inorganic fertilizer-N application through supplementation by biological nitrogen fixation. This could be a new and important source of nitrogen biofertilizer in the early phase of oil palm cultivation in the field.

Oil palm (*Elaeis guineensis* Jacq.) thrives well in Malaysia and many other countries in Southeast Asia and Africa. With Malaysia presently ranked second in the world after Indonesia as a producer of palm oil, the commodity is a major contributor to the country’s economic development. Oil palm in Malaysia occupied a planted area of 4.69 million hectares in 2009, or about two-thirds of the total land area under agriculture (http://econ.mpob.gov.my/economy/Overview_2009.pdf). The crop is commonly cultivated in tropical soils, which are normally deficient in nitrogen. Young oil palms are badly affected by the insufficiency of nitrogen, which causes yellowing of the leaves, and eventually necrosis (9). The excessive use of nitrogenous fertilizer for oil palm planting, however, could pose a hazard to the agro-environment. Such practice would also increase oil palm management costs, making the industry less profitable and hence less attractive. Diazotrophic plant growth-promoting rhizobacteria (PGPR) may hold the key to the availability of nitrogen fertilizers that are cost-effective while being environmentally friendly. Biological N_2_ fixation (BNF) is recognized as an important component of the nitrogen cycle in a range of ecosystems, including several extreme environments (6, 8, 13, 24). Several PGPRs, including strains of *Azospirillum*, *Azotobacter*, *Bacillus* and *Herbaspirillum*, have been reported to exert a beneficial effect upon the plant growth of many crops, such as cotton (22), maize (3), sugarcane (27), rice (19) and oil palm (1).

Several methods are used to assess the ability of PGPRs to fix atmospheric nitrogen. In this regard, the techniques based on ^15^N isotope dilution are versatile and can be adapted to various experimental situations (15, 23). Using the ^15^N isotope dilution approach, N_2_ fixation by diazotrophic bacteria has been shown to contribute up to 20–50% of the total oil palm seedling N requirements under glasshouse conditions (2). Inoculation of these bacteria into the rhizosphere also improves nutrient accumulation of oil palm seedlings under field nursery conditions (1). Using the ^15^N natural abundance technique, de Carvalho and co-workers showed the high potential for N_2_ fixation to benefit some oil palms in the nursery where nitrogen derived from atmosphere (NDFA) could reach 50% of the plant’s requirement (14). *Bacillus sphaericus* UPMB-10, a rhizobacterium isolated from oil palm roots in Malaysia, has been shown to be a potential biofertilizer-providing microorganism with the ability to contribute 28% of the plant’s total nitrogen requirement through atmospheric nitrogen fixation (2).

As an extension of the earlier findings by Amir *et al.*(1, 2), this is the first study on oil palm that reports the extent of uptake of N derived from N_2_ fixation by the PGPR, *Bacillus sphaericus* UPMB-10, under field conditions, using the ^15^N isotope dilution method.

## Materials and Methods

**Fig. 1:**
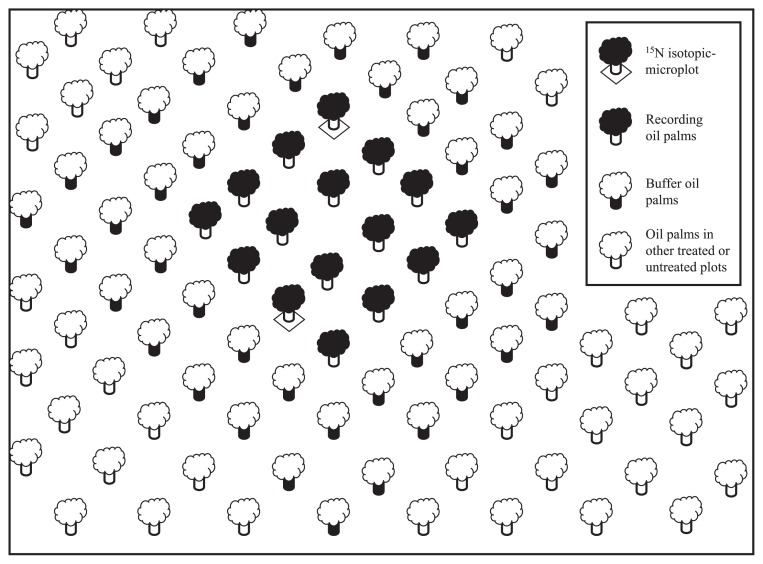
A plot with 16 recording oil palms (including 2 randomly selected palms receiving ^15^N-labeled fertilizer) and two outermost rows serving as a buffer. The buffer oil palms help to prevent cross-contamination between plots. They were treated the same as the recording palms in the ^15^N isotopic microplot. Recording palms were also used to conduct vegetative growth measurements (data not presented in the present study).

The experiment was conducted in a field at Tangkah Estate, Sime Darby Plantation Berhad (formerly Golden Hope Plantation Berhad), Tangkak, Johor, in southern Peninsular Malaysia (2°21′ N, 102°40′ E). Some chemical properties of the 0–15 cm layer of the Bungor soil (Ultisol) in the experimental area are presented in Table 1. Fourteen-month-old GH500 cloned oil palms were allowed to establish for 5 months after transplantation in the field in a triangular planting pattern of 8 m by 8 m (Fig. 1). The upkeep and maintenance of the trial plots included a normal estate manuring schedule of inorganic straight fertilizers, comprising N as ammonium sulfate, P as Christmas Island Rock Phosphate, K as muriate of potash, Mg as kieserite and B as borate (17). Straight fertilizer is a fertilizer that contributes a single nutrient to the crops.

*Bacillus sphaericus* UPMB-10, isolated in Malaysia from oil palm roots (2), was subcultured on tryptic soy agar (TSA) (Merck KGaA Germany) to produce a pure mother culture for inoculum production. The carrier-based inoculum of UPMB-10 strain was prepared by transferring 1.0 mL of a 24-h culture (≥1×10^9^ cfu mL^−1^) to 100 mL tryptic soy broth (TSB) (Merck KGaA) contained in 500 mL flasks. The flasks were incubated on an orbital shaker at 28±2°C for 24 hours and the number of viable cells in the culture was determined by the spread-plate method on TSA after 24-h incubation. An aliquot (30 mL) of broth culture was withdrawn from the flask when the cell concentration reached 10^9^ cfu mL^−1^ and was injected through a puncture into a double layered gamma-irradiated (50 kGy) polyethylene bag containing a mixture of (a) ground oil palm frond with the following compositions: 492 mg kg^−1^ total N, 54 mg kg^−1^ available P, 30 mg kg^−1^ available K, 4 mg kg^−1^ available Ca and 1 mg kg^−1^ available Mg, and (b) a commercial peat-based organic fertilizer with the following compositions: 253 mg kg^−1^ total N, 389 mg kg^−1^ available P, 172 mg kg^−1^ available K, 536 mg kg^−1^ available Ca and 126 mg kg^−1^ available Mg. The polyethylene bag contained 1,500 g of (a) and 500 g of (b) in a homogenous mixture. The inoculated bags were incubated at room temperature (28±2°C) for 2 weeks before use. The cultures were then checked for quality by the spread-plate method. The minimum population of strain UPMB-10 was ≥10^9^ cfu g^−1^ during field inoculation.

In the field, the plants were laid down in randomized complete block design with 4 treatments and 4 replicates, as shown in Table 2. The (Uninoculated−N_i_+^15^N_i_) and (Uninoculated+100% N_i_+^15^N_i_) treatments served as negative and positive controls, respectively, and also as a benchmark for deficient N (negative control) and optimum N (positive control). The (Inoculated+67% N_i_+^15^N_i_) treatment involved inoculation with *B. sphaericus* strain UPMB-10 inoculum. The (Uninoculated+67% N_i_+^15^N_i_) control treatment had similar N_i_ (67%) to the inoculated treatment. All uninoculated treatments were provided with killed inoculum (gamma-irradiated at 50 kGy) per palm. ‘100% N_i_’ and ‘67% N_i_’ refer to the full recommended inorganic N fertilizer regime (17) and 67% of the full N fertilizer regime, respectively.

Recordings were made from 16 palms for each of the 16 plots (4 treatments by 4 replicates). Palms in the two outermost rows served as a buffer (Fig. 1). The ^15^N-labeled fertilizer used was (^15^NH_4_)_2_SO_4_ (ammonium sulfate) with 10.13 atom % ^15^N excess (at.%^15^N_e_) serving as a tracer. The field experiment was initiated by the application of ^15^N-labeled fertilizer 5 months after transplanting. Within the 16 recording palms, 2 palms (micro-plot) received labeled ^15^N, with 10.13 at.%^15^N_e_ ammonium sulfate at a rate of 1 g N m^−2^ (Fig. 1). The ^15^N-labeled fertilizer was uniformly applied in liquid form using 2 L distilled water per isotopic plot of 1 m^2^ size. The plots were then covered with black polythene sheets evenly to reduce ^15^N-labeled fertilizer loss. A week later, the black polythene sheets at the ^15^N isotopic microplots were removed after the inner surface of each sheet was rinsed with water prior to inoculum application (31). The black polythene sheets were used once only for all inoculated and uninoculated ^15^N isotopic microplots. Inoculum for the first inoculation was then applied followed by the second inoculation four months later. The (Inoculated+67% N_i_+^15^N_i_) treatment was carried out at a rate of 2 kg inoculum (containing more than 10^9^ cfu g^−1^*B. sphaericus* UPMB-10) by raking the surface of soil to a depth of approximately 5 cm within an area of 1 m^2^, and at a rate equivalent to 296 kg ha^−1^.

Harvesting was carried out 240 days (8 months) after the ^15^N-labeled fertilizer application. Four palms from each treatment were harvested destructively, and separated into leaflets, rachis, stem and roots. The major roots were extracted with a backhoe tractor, and the remaining roots were excavated by shoveling and sieving the soil within the area occupied by the harvested palm. Fresh weights and weights of oven-dried (70°C for 72 h) sub-samples were recorded. Samples were ground to pass through 0.5 mm sieves and analyzed for total N by the semi-micro Kjeldahl method (5) and atom %^15^N excess using the NOI-6PC emission spectrometer at Malaysian Nuclear Agency, Bangi, Selangor, Malaysia. The ^15^N abundance found in palm tissue was corrected for the atom %^15^N excess present in the atmosphere (0.3663 at.%^15^N_e_) (30).

N_2_ fixation in the whole palm was calculated from weighted atom excess (WAE) in the inoculated palm (Inoculated+67% N_i_+^15^N_i_) and uninoculated palm (Uninoculated+67% N_i_+^15^N_i_), using the following formula (15):

$$\text{WAE}=\frac{\text{AE}(\text{Lf})\times \text{TN}(\text{Lf})+\text{AE}(\text{Rc})\times \text{TN}(\text{Rc})+\text{AE}(\text{St})\times \text{TN}(\text{St})+\text{AE}(\text{Rt})\times \text{TN}(\text{Rt})}{\text{TN}\;(\text{Lf}+\text{Rc}+\text{St}+\text{Rt})}\times 100$$

where AE, TN, Lf, Rc, St and Rt are atom % ^15^N excess, total N, leaflets, rachis, stems and roots, respectively.

The % of N derived from atmospheric N (%Ndfa) was then calculated as follows:

$$\%\text{Ndfa}=1-\frac{\text{WAE in inoculated oil palm}}{\text{WAE in uninoculated oil palm}}\times 100$$

The data were statistically analyzed using a linear contrast one-way analysis of variance (ANOVA) followed by Dunnett’s test (SPSS Statistics version 17.0). The data were tested for significance of differences between the experimental treatments.

## Results

### Dry matter yield

The inoculated palms (Inoculated+67% N_i_+^15^N_i_) accumulated the highest total dry matter at 10.5 kg palm^−1^ followed by uninoculated palms fertilized with the full rate of N (Uninoculated+100% N_i_+^15^N_i_) at 8.4 kg palm^−1^, and uninoculated palms fertilized with 67% inorganic-N (Uninoculated+67% N_i_+^15^N_i_) at 8.3 kg palm^−1^ (Table 3). Among the plant parts, the dry weight of the rachis and leaflets increased significantly (*p*<0.05) after inoculation with *B. sphaericus* UPMB-10, as compared with uninoculated palms supplied with 67% inorganic N fertilizer. Rachis accounted for the highest proportion of dry matter, making up nearly 40% of the total weight. This was followed by leaflets (27%), stems (16%) and roots (14%). It was evident from this that over the 240 days of the trial, the growth of the young immature oil palms in field occurred predominantly aboveground, especially in the rachis and leaflets.

### Total nitrogen yield

The amount of N present in young immature oil palm on average was 95 g palm^−1^ (Table 4), with the distributions among plant parts being 60% in leaflets, 15% in rachis, 16% in stem and 9% in roots. On average, the palms inoculated with *B. sphaericus* UPMB-10 (Inoculated+67% N_i_+^15^N_i_) accumulated more N at 125 g palm^−1^ than uninoculated palms (Uninoculated+67% N_i_+^15^N_i_) at 90 g palm^−1^, although this difference was not statistically significant. Among the palm parts, leaflets and rachis accumulated significantly higher amounts of N (*p*<0.05) at 75 g palm^−1^ and 19 g palm^−1^ respectively, than uninoculated palms, where N contents were 53 g palm^−1^ and 12 g palm^−1^, respectively.

### Uptake of labeled nitrogen

The weighted %^15^N atom excess for the reference palms (Uninoculated+67% N_i_+^15^N_i_) was 0.065 at.%^15^N_e_. This was significantly higher (*p*<0.05) than 0.023 at.%^15^N_e_ obtained for the inoculated palms (Inoculated+67% N_i_+^15^N_i_) after 240 days in the field. Among plant parts, leaflets of inoculated palms showed significantly lower ^15^N enrichment at 0.021 at.%^15^N_e_ than uninoculated palms, which gave an average reading of 0.091 at.%^15^N_e_. Rachis, stems and roots on average also had lower but insignificant ^15^N enrichment at 0.030, 0.017 and 0.031 at.%^15^N_e_ respectively, than at 0.034, 0.019 and 0.034 at.%^15^N_e_, respectively for uninoculated oil palms (Table 5).

### Nitrogen fixation

On the basis of the whole palm, inoculation with UPMB-10 strain had 63.4%Ndfa (78.1 g N fixed palm^−1^), which is equivalent to 17.4 kg N ha^−1^ year^−1^, at a planting density of 148 palms ha^−1^ (Table 6). The mean %Ndfa in different plant parts was 74.7, 12.7, 13.2 and 13.4 for leaflets, rachis, stems and roots, respectively (based on the weighted atom % ^15^N excess values). Among the plant parts, the %Ndfa in inoculated palm leaflets accumulated the highest fixed N (74.7%Ndfa or 55.4 g palm^−1^, equivalent to 12.3 kg N ha^−1^ year^−1^, at a planting density of 148 palms ha^−1^).

## Discussion

Many researchers consider ^15^N isotope dilution to be the most accurate technique to quantify biological N_2_ fixation by plants under greenhouse and nursery conditions, and even in the field (2, 11, 12, 20, 25). In our study, the atom % ^15^N excess in the whole young oil palm inoculated with *B. sphaericus* UPMB-10 (Inoculated+67% N_i_+^15^N_i_) was significantly lower than in uninoculated palms (Uninoculated+67% N_i_+^15^N_i_), thus indicating that substantial N_2_ fixation occurred. *B. sphaericus* UPMB-10 inoculation resulted in the acquisition of N from the atmosphere, which contributed 63% (almost two-thirds) of the plant N uptake. This was equivalent to 78 g N fixed palm^−1^ in the 240 day period of growth or 11.6 kg N ha^−1^, based on a planting density of 148 palms ha^−1^. The findings in this study support the previous work of Amir *et al.*(2), but the 63% proportional contribution of fixed N was even higher than the 28% they had earlier reported. The higher contribution of N by *B. sphaericus* UPMB-10 inoculation through BNF in the present study could have resulted from the use of solid inoculum application, a strategy adopted after studying the outcomes and suggestions from the previous work of Amir *et al.*(1, 2). In the present study, field inoculation was conducted using solid substrate inoculum, not as in the earlier reports (1, 2), which used liquid inoculum. The solid substrate inoculum has several added benefits over liquid inoculum as it provides a substantial amount of carbon source, which was lacking in the liquid inoculum. In addition, the solid substrate inoculum was incubated for a minimum of two weeks to allow the inoculum population to reach 1×10^9^ cfu g^−1^ substrate. Thus, the inoculum in the inoculated treatment in young oil palms would have a high population of *B. sphaericus* UPMB-10 and more supply of organic substrate to sustain growth and multiply in the soil, and subsequently offer more Ndfa (63%) through N_2_-fixing activity. The enhancement of N_2_-fixing activity could be due to a higher concentration of carbon sources; namely, starch, hemicellulose and degradation products from beneficial soil microbial interactions (28). In addition, the role of solid substrate in supplying an organic carbon source and other degradation products could also provide ‘shelter’ for bacterial cells from direct sunlight and as a buffer zone for solid substrate-soil surface interactions prior to rhizosphere colonization.

Among the plant parts, the accumulation of N in leaflets significantly increased in inoculated oil palms in parallel with the respective increases in %Ndfa values as compared with the uninoculated control palms. This could be related to the high amount of N nutrition required in the leaves for photosynthesis, especially crucial in the early growth stages of oil palm in the field (10).

In this present study, the roots contained 13% of the total N_2_ fixed by the whole palm and 14% of the accumulated dry matter. This relatively small proportion of the roots in relation to the whole plant was similar to the 12% reported by Carranca *et al.*(7) in pea roots and 8–15% reported by Samba *et al.*(25) for legume roots of *Crotalaria* species. In both of these studies, roots were omitted from their ^15^N evaluations. Nevertheless, due caution is advisable since ignoring the contribution from roots might result in significant underestimation of N_2_ fixed in some plants, as has been reported in *Acacia* sp., *Faidherbia albida*, *Gliricidia sepium*, *Leucaena leucocephala* and *Pterocarpus* sp. (18, 21, 26, 29). Nevertheless, this does not appear to be the case for oil palm, which is a non-nodulating and non-leguminous crop. Conversely, much of the total dry matter was accumulated aboveground.

The present study indicated that inoculation with *B. sphaericus* UPMB-10 significantly improved N and dry matter yields of leaflets and rachis of young immature oil palms 8 months after treatment, although this effect was not observed in the stem or roots. The leaflets and rachis together accounted for most of the N and dry weight accumulation of the plants. To secure compelling evidence of biological N_2_ fixation, Boddey (4) maintained that associative biological N_2_ fixation should include both higher N yield and lower ^15^N enrichment of the inoculated plant compared to the uninoculated plant. A significant dry matter or fruit yield increase due to inoculation with diazotrophic organisms should be accompanied by such evidence before it can be confidently concluded that the response is due to N_2_ fixation, and not to some other factors. The present study, which showed both higher N yield and lower ^15^N enrichment as a result of soil inoculation with *B. sphaericus* UPMB-10, is in agreement with the criteria suggested by Boddey (4). What was still left to be demonstrated was an increase in fruit yield. Since immature oil palms were used in the present study, the effect of inoculation on the fruit yield was outside the scope of the present study. Nevertheless, nutritional, growth and BNF measurements up to palm maturity (2.5 years after planting) and fruit-bearing stages (3 years after planting) in future studies would provide a clearer picture of the effect of *B. sphaericus* UPMB-10 inoculation on oil palm cultivation. In addition, the planting density, soil type and trial location would need to be carefully considered in order to reduce the soil and spatial variability factors that could compromise data accuracy (14, 16).

In conclusion, field planting of young immature oil palm in soil inoculated with *Bacillus sphaericus* UPMB-10 reduced N fertilizer dependence through the supplementation of N by biological nitrogen fixation. The inoculation process contributed N_2_ fixation from the atmosphere, which provided 63%, or nearly two-thirds of the total N uptake of the young immature oil palm. Hence, *B. sphaericus* UPMB-10 has the potential to be formulated for use as a biofertilizer. It is proposed that the experimental period should be extended in future studies until the flowering and fruiting stages of the oil palm to elucidate further the role of the diazotrophic rhizobacterium *B. sphaericus* UPMB-10 in promoting the growth and fruit yield of oil palm.

## Figures and Tables

**Table 1:** Chemical properties of the soil^2^ (Ultisol) from the oil palm experimental field in Tangkak, Johor

pH (KCl) (1:2.5)	mg kg^−1^				

	Total N	Available			

		P^1^	K	Ca	Mg
4.7	16.0	22.3	19.0	55.0	15.0

1 Extracted with an aqueous solution of 0.05 M HCl and 0.0125 M H_2_SO_4_

2 Bungor sandy clay loam soil, with 1.2% total C content

**Table 2:** Treatment design and description of biological nitrogen fixation by inoculated PGPR *Bacillus sphaericus* UPMB-10 in association with the growth of immature oil palm

Treatments	Descriptions
Uninoculated-N_i_+^15^N_i_	No inorganic-N fertilization, with no inoculation, supplemented with 10.13 atom %^15^N excess of inorganic-N-labeled fertilizer.
Uninoculated+67% N_i_+^15^N_i_	Inorganic normal N fertilizer (unlabeled ammonium sulfate) applied at 67% standard estate rate, with no inoculation, supplemented with 10.13 atom %^15^N excess of inorganic-N-labeled fertilizer.
Inoculated+67% N_i_+^15^N_i_	Inorganic normal N fertilizer (unlabeled ammonium sulfate) applied at 67% standard estate rate, with *B. sphaericus* strain UPMB-10 inoculation, supplemented with 10.13 atom %^15^N excess of inorganic-N-labeled fertilizer.
Uninoculated+100% N_i_+^15^N_i_	Inorganic normal N fertilizer (unlabeled ammonium sulfate) applied at full (100%) standard estate rate, with no inoculation, supplemented with 10.13 atom %^15^N excess of inorganic-N-labeled fertilizer.

Note: All treatments were labeled with ^15^N-labeled ammonium sulfate (10.13 atom %^15^N enrichment as a tracer) according to 1 g N m^−2^.

**Table 3:** Dry matter yield (% of total dry matter) and its distribution in plant parts of field-grown immature oil palm

Treatments	Dry matter yield (kg palm^−1^) Mean±SEM				Total dry matter yield (kg palm^−1^)	Total dry matter yield (kg ha^−1^)^b^

	Leaflets	Rachis	Stems	Roots		
Uninoculated-N_i_+^15^N_i_	1.8±0.2 (27.8)^a^	2.6±0.3 (40.2)	1.1±0.1 (16.4)	1.0±0.2 (15.6)	6.5	968
Uninoculated+67% N_i_+^15^N_i_	2.0±0.2 (24.3)	3.6±0.4 (43.3)	1.3±0.2 (15.9)	1.4±0.1 (16.5)	8.3	1,228
Inoculated+67% N_i_+^15^N_i_	2.8±0.5* (26.4)	4.7±0.7* (44.9)	1.7±0.3 (15.7)	1.4±0.1 (13.0)	10.5	1,557
Uninoculated+100% N_i_+^15^N_i_	2.2±0.2 (26.5)	3.7±0.3 (44.4)	1.3±0.1 (15.7)	1.1±0.2 (13.4)	8.4	1,245

a values in parentheses represent % of total dry matter.

b 1 ha=148 palms

* *p*<0.05 versus control (Uninoculated+67% N_i_+^15^N_i_) (One-way ANOVA and Dunnett’s *post-hoc* test)

**Table 4:** Total N yield (% of total N) and its distribution in immature oil palm

Treatments	N yield (g palm^−1^) Mean±SEM				Total N yield

	Leaflets	Rachis	Stem	Roots	
Uninoculated − N_i_ + ^15^N_i_	40.4±5.7 (66.3)^a^	8.3±1.1 (13.7)	7.0±0.6 (11.5)	5.8±1.2 (8.5)	61.0
Uninoculated + 67% N_i_ + ^15^N_i_	53.4±5.9 (59.3)	12.1±1.4 (13.4)	15.4±2.1 (17.1)	9.1±1.0 (10.2)	90.0
Inoculated + 67% N_i_ + ^15^N_i_	75.0±12.6* (60.2)	18.9±4.5* (15.2)	20.6±3.9 (16.6)	10.0±0.8 (8.0)	124.5
Uninoculated + 100% N_i_ + ^15^N_i_	57.0±5.3 (54.8)	19.9±3.3* (19.1)	18.9±1.7 (18.2)	8.2±1.7 (7.9)	104.0

a values in parentheses represent % of total N in palm

* *p*<0.05 versus control (Uninoculated+67%N_i_+^15^N_i_) (One-way ANOVA and Dunnett’s *post-hoc* test)

**Table 5:** Distribution of %^15^N atom excess in different plant parts and the mean weighted atom excess (WAE) for the whole plant

Treatments	% ^15^N atom excess (Mean±SEM)				WAE (whole palm)

	Leaflets	Rachis	Stems	Roots	
Uninoculated-N_i_+^15^N_i_	0.048±0.0028	0.061±0.0045	0.041±0.0046	0.069±0.0067	0.051±0.0033
Uninoculated+67% N_i_+^15^N_i_	0.091±0.0159	0.034±0.0019	0.019±0.0017	0.034±0.0043	0.065±0.0089
Inoculated+67% N_i_+^15^N_i_	0.021*±0.0003	0.030±0.0032	0.017±0.0021	0.031±0.0019	0.023*±0.0009
Uninoculated+100% N_i_+^15^N_i_	0.015*±0.0006	0.024±0.0031	0.030±0.0047	0.037±0.0039	0.021*±0.0011

* *p*<0.05 versus control (Uninoculated+67% N_i_+^15^N_i_) (One-way ANOVA and Dunnett’s *post-hoc* test).

Note: (Uninoculate-N_i_+^15^N_i_) and (Uninoculated+100% N_i_+^15^Ni) are negative (no nitrogen fertilizer application) and positive controls (full nitrogen fertilizer application at estate recommended rate), respectively. All treatments were labeled with ^15^N_i_ as a tracer to quantify biological nitrogen fixation using indirect ^15^N isotope dilution technique.

**Table 6:** Estimates of proportions (%Ndfa) and amounts, g N palm^−1^* of N fixed in the whole plant and in the different plant parts of field grown immature oil palm, with *Bacillus sphaericus* UPMB-10 inoculation

%Ndfa (amount of N^2^ fixed, g palm^−1^) Mean±SEM				

Whole palm	Leaflets	Rachis	Stems	Roots
63.4±4.5	74.7±3.6	12.7±7.9	13.2±6.9	13.4±8.4
(78.1±11.6)	(55.4±8.3)	(3.0±1.9)	(2.5±1.0)	(1.5±1.0)

* In parentheses
